# Protective Effects of *Bacillus subtilis* HH2 against Oral Enterotoxigenic *Escherichia coli* in Beagles

**DOI:** 10.3390/vetsci10070432

**Published:** 2023-07-03

**Authors:** Jinpeng Yang, Xinyue Zhang, Ziyao Zhou, Caiwu Li, Run Luo, Haifeng Liu, Hualin Fu, Zhijun Zhong, Liuhong Shen, Suizhong Cao, Yan Luo, Desheng Li, Guangneng Peng

**Affiliations:** 1 Key Laboratory of Animal Disease and Human Health of Sichuan Province, College of Veterinary Medicine, Sichuan Agricultural University, Chengdu 611130, China; 2 China Conservation and Research Center for the Giant Panda, Key Laboratory of State Forestry and Grassland Administration on Conservation Biology of Rare Animals in the Giant Panda National Park, Chengdu 610083, China

**Keywords:** *Bacillus subtilis*, enterotoxigenic *Escherichia coli*, gut microbiome, intestinal barrier, probiotic, beagles

## Abstract

**Simple Summary:**

Enterotoxigenic *Escherichia coli* (ETEC) is an important pathogen that causes diarrhea in both humans and animals, thereby posing a serious threat to public health and animal agriculture. *Bacillus subtilis*, a probiotic, offers a novel approach to reduce the need for antibiotics and plays a crucial role in treating various intestinal diseases. We previously isolated a strain of *B. subtilis* HH2 from giant panda feces, which has shown multiple beneficial functions in vitro and in vivo. However, studies on the protective effect of *B. subtilis* on companion animals with orally administered ETEC have not been reported. Therefore, we explored the effects of *B. subtilis* HH2 on the fecal microbiota, intestinal barrier integrity, and non-specific immunity in beagles challenged with ETEC. Experimental results showed that *B. subtilis* HH2 could alleviate diarrhea caused by ETEC, improve non-specific immunity and intestinal barrier integrity, and modulate gut microbiota. Notably, more indicators are needed to determine its protective effect on beagles in future studies.

**Abstract:**

This study evaluated the protective effect of *Bacillus subtilis* HH2 on beagles orally challenged with enterotoxigenic *Escherichia coli* (ETEC). We assessed the physiological parameters and the severity of diarrhea, as well as the changes in three serum immunoglobulins (IgG, IgA, and IgM), plasma diamine oxidase (DAO), D-lactate (D-LA), and the fecal microbiome. Feeding *B. subtilis* HH2 significantly reduced the severity of diarrhea after the ETEC challenge (*p* < 0.05) and increased serum levels of IgG, IgA, and IgM (*p* < 0.01). *B. subtilis* HH2 administration also reduced serum levels of DAO at 48 h after the ETEC challenge (*p* < 0.05), but no significant changes were observed in D-LA (*p* > 0.05). Oral ETEC challenge significantly reduced the richness and diversity of gut microbiota in beagles not pre-fed with *B. subtilis* HH2 (*p* < 0.05), while *B. subtilis* HH2 feeding and oral ETEC challenge significantly altered the gut microbiota structure of beagles (*p* < 0.01). Moreover, 14 days of *B. subtilis* HH2 feeding reduced the relative abundance of *Deinococcus*-*Thermus* in feces. This study reveals that *B. subtilis* HH2 alleviates diarrhea caused by ETEC, enhances non-specific immunity, reduces ETEC-induced damage to the intestinal mucosa, and regulates gut microbiota composition.

## 1. Introduction

*Escherichia coli* is thought to be a natural component of the gut microbiota that lives in the intestinal tracts of both humans and animals. However, among them, enterotoxigenic *Escherichia coli* (ETEC) can cause diarrhea in young animals, especially newborn piglets, calves, lambs, and weaned piglets, causing enormous economic losses to the breeding industry [1]. In veterinary practice, antibiotics are widely used to treat and prevent gastrointestinal diseases, but antibiotic resistance and drug residues due to antibiotic misuse pose a potential risk to public health safety. According to the latest estimates, 4.95 million human deaths in 2019 were associated with bacterial resistance, with 1.27 million of those deaths attributed to bacterial resistance [2]. Therefore, several strategies have been implemented to reduce antibiotic use and antibiotic resistance in animals, such as antibiotic usage regulation, veterinary guidance and training, and alternative treatment methods. Of these, maintaining gastrointestinal health will be an important factor in reducing antibiotic misuse while also improving livestock health and productivity, including growth and reproductive performance, as well as the production and quality of meat, eggs, and milk [3].

Currently, probiotics as microbial food supplements have been widely used to maintain gut health as they promote the digestion and absorption of nutrients, maintain the balance of the gut microbial community, inhabit harmful pathogens, strengthen the intestinal barrier, and enhance immunity [4,5]. Probiotic *Bacillus subtilis* as an alternative to antibiotics has been receiving a lot of attention due to its desirable characteristics. Studies have shown that *B. subtilis* can regulate gut microbiota composition by enriching potentially beneficial bacteria and inhibiting pathogenic bacteria [6,7,8]. In vitro studies also found that *B. subtilis* displayed an inhibitory effect against ETEC, *Staphylococcus aureus*, and *Salmonella*, either by direct contact or by indirect effect [9,10,11]. Moreover, *B. subtilis* has the ability to increase serum immunoglobulins [7], enhance phagocytosis and lysozyme activity [12], and stimulate the growth and development of immune organs to improve animal immunity [13]. In addition, *B. subtilis* exerts cytoprotective effects by influencing processes such as inflammatory response, gene expression related to the intestinal barrier, oxidative stress, and apoptosis [6,14,15,16]. Currently, numerous studies have shown that *Bacillus* spp. exhibit inhibitory effects on ETEC both in vivo and in vitro. Specifically, *B. subtilis* has been found to hinder the growth and colonization of ETEC within the gut through multiple mechanisms, such as producing antimicrobial compounds [17], modulating the gut microbiota [18], and strengthening the gut barrier function [19,20]. In vitro studies have shown that *B. subtilis* reduces ETEC adhesion to the intestinal cell line while inhibiting ETEC-induced phosphorylation of extracellular signal-regulated kinases 1 and 2 [20,21]. Additionally, *B. subtilis* can also modulate animal immunity, improve performance, and reduce the incidence of diarrhea in ETEC-challenged animals [19,22]. Overall, *B. subtilis* has promising applications in the treatment of ETEC infections, but its benefits need further validation, especially for different model animals.

We previously isolated the novel probiotic *B. subtilis* HH2 from the feces of healthy captive giant pandas and observed its antipathogenic effects in vitro and in its alleviation of colitis in vivo [23,24,25]. At the transcriptional level, *B. subtilis* HH2 triggers adaptive mechanisms, indicating its potential as a probiotic for pandas fed a high-fiber diet. Additionally, we found that the surfactin secreted by *B. subtilis* HH2 can inhibit *E. coli*, and *B. subtilis* HH2 can also ameliorate TNBS-induced colitis in a rabbit model by modulating gut microbiota composition and improving intestinal barrier function. However, little is known about the impact of *B. subtilis* HH2 on physiological parameters, non-specific immunity, gut microbiota, and gut barrier function in beagles infected with ETEC. Therefore, to further evaluate the in vivo effect of *B. subtilis* HH2 on ETEC, we used beagles as a model of ETEC infection to study the effects of *B. subtilis* HH2 strains on clinical indicators (rectal temperature, respiratory rate, heart rate, and severity of diarrhea), the immunoglobulin (IgA, IgG, and IgM), integrity of intestinal barrier, and fecal microbiota.

## 2. Materials and Methods

### 2.1. Ethics Statement

The animal experiments conducted in this study were performed in adherence to the approved guidelines for the care and use of laboratory animals by the Institutional Animal Care and Use Committee of Sichuan Agricultural University, located in Sichuan, China (2021203070).

### 2.2. Preparation of Probiotic Bacterial Strain

*B. subtilis* HH2 was isolated from fresh feces of healthy giant pandas at the Bifengxia Base of China Conservation and Research Center for the Giant Panda in Ya’an, Sichuan Province, China. ETEC K88 strain was purchased from Suzhou Beina Chuanglian Biotechnology Co., Ltd. (Suzhou, China). *B. subtilis* HH2 was inoculated in 100 mL of Luria-Bertani (LB) broth and incubated for 8–10 h at 37 °C with shaking at 150 rpm. Then, the actual concentration of the bacterial solution was calculated by the flat colony counting method. Finally, the bacterial solution was resuspended using sterile saline to a final concentration of 5.0 × 10^8^ CFU/mL, and then 2.0 × 10^9^ CFU/mL of ETEC bacterial suspension was prepared as above.

### 2.3. Animal and Experimental Design

A total of 11 healthy beagles (body weights: 4.5 ± 0.5 kg, 3–4 months old, born in the same litter) were purchased from Chengdu Dashuo Experimental Animal Co., Ltd. (Chengdu, China) and randomly divided into two groups: five dogs in the control group (dogc) and six dogs in the treatment group (dogt). After vaccination and deworming, the dogs were individually housed in cages measuring 85 cm in length, 60 cm in width, and 70 cm in height, located in the kennels of the College of Veterinary Medicine at Sichuan Agricultural University. They had unrestricted access to fresh water throughout the experiment. The cages were cleaned once a day using compound sodium hypochlorite disinfectant and kept dry. The room temperature was maintained at 16 to 28 °C (12 h light and 12 h dark per day). During the adaptation period, the dogs were provided with 2 h of outdoor walking time daily to facilitate acclimatization. In addition, they received daily indoor walking time of 2 h to promote their physical and mental well-being during feeding experiments. After the confirmation of their health and a one-month adaptation period, all dogs were assigned to feeding experiments based on previous studies [26]. The dogc group was fed a commercial dog food (Paitejia Natural All-Life Stage Dog Food, HebeiYoujie Pet Food Co., Ltd., Hebei, China; Supplementary Table S1) twice a day for 17 days. The dogt group was provided the same commercial dog food with additional oral administration of 10 mL of *B. subtilis* HH2 (5.0 × 10^8^ CFU/mL) for two weeks. On day 15, both dogt and dogc groups were orally administered 10 mL of ETEC K88 strain (2.0 × 10^9^ CFU/mL). This dosage was determined based on preliminary studies to induce only mild loss of appetite or diarrhea in the dogs [27]. Anal swabs were collected from both groups before the oral administration of *B. subtilis* HH2 and at 0 h, 24 h, 48 h, and 72 h after the oral administration of ETEC K88 strain, and were stored at −80 °C for genomic DNA extraction. Meanwhile, blood samples were collected from the cephalic vein at 0 h, 24 h, 48 h, and 72 h after the oral administration of ETEC K88 strain. At the end of the experiment, a comprehensive examination was conducted, revealing that all dogs were in excellent health, and subsequently, they were successfully adopted by local residents.

### 2.4. Clinical Investigations

To assess the health status of the dogs and the effect of the intervention, the respiratory rate, rectal temperature, heart rate, and severity of diarrhea were recorded daily throughout the experiment period. Rectal temperature testing was performed by checking the dog’s rectal temperature with a mercury thermometer. The respiratory rate and heart rate were determined by observing the number of the dog’s chest rises and falls and auscultating through a stethoscope for one minute, respectively, while they were resting or sleeping. In this study, the normal physiological parameters provided in the Merck Veterinary Manual (2016) for dogs were used as a reference to establish the normal clinical values. In addition, all dogs were monitored daily for activity, appetite, vomiting, frequency of diarrhea, fecal consistency, and dehydration, which would be used to assess the severity of diarrhea. The scoring criteria for the degree of canine diarrhea were referenced from the scoring system proposed by Albert E. Jergens and S. Unterer et al. for the assessment of canine inflammatory bowel disease activity [28,29]. The severity of diarrhea is indicated by four 4 levels: normal, mild, moderate, and severe, represented by the number ranges 0–3, 4–5, 6–8, and 9 or greater than 9, respectively.

### 2.5. Indicator Detection of Intestinal Immunity and Intestinal Barrier

After sampling, blood samples were clotted at room temperature and centrifuged at 2000× *g* for 20 min at 4 °C to obtain serum. The serum samples were pipetted out with a pipette and stored in 1.5 mL polypropylene tubes at −80 °C before analysis. The levels of serum IgA, IgM, IgG, and D-lactate (D-LA) and diamine oxidase (DAO) activity were analyzed by enzyme-linked immunosorbent assay kits (Beijing DG Biotechnology Co., Ltd., Beijing, China) following the manufacturer’s instructions.

### 2.6. DNA Extraction and 16S rRNA Gene Sequencing

Stool samples collected via anal swab were suspended in sterile saline and subjected to DNA extraction using the Stool Microbiome DNA Extraction Kit (MGI Tech Co., Ltd., Shenzhen, China) following the manufacturer’s instructions. The concentration and quality of DNA in each sample were assessed using NanoDrop (Thermo Fisher Scientific, Waltham, MA, USA) and 1% agarose gel electrophoresis, respectively. The extracted DNA was then diluted to 1 ng/μL with sterile water and used as the template for PCR amplification of the bacterial 16S rDNA V3-V4 region using primers F (5′-AGAGTTTGATCCTGGCTCAG-3′) and R (5′-TACGGCTACCTTGTTACGACTT-3′). The PCR reaction contained 2 µL of template DNA, 12.5 µL of Taq PCR Master Mix, 1 µL of each primer, and 8.5 µL of nuclease-free double-distilled water in a total volume of 25 µL. Cycling conditions included an initial denaturation step at 94 °C for 5 min, followed by 30 cycles of 94 °C for 1 min, 55 °C for 5 min, 72 °C for 90 s, and a final extension step at 72 °C for 8 min. The PCR product was purified using a gel recovery kit from Qiagen. Subsequently, the resulting library was prepared using the TruSeq^®^ DNA PCR-Free Sample Preparation Kit (Illumina, USA). The library was then quantified using Qubit and quantitative PCR. Finally, sequencing was performed using HiSeq2500 PE250 after meeting the quality control criteria.

### 2.7. Bioinformatic Analysis

To process the sample data, paired-end reads were first split by barcode and primer sequences. FLASH (V1.2.7, http://ccb.jhu.edu/software/FLASH/, accessed on 13 April 2022) was used to splice the paired-end reads into Raw Tags, after removing barcode and primer sequences [30]. The Clean Tags were obtained by applying the quality control steps outlined in Qiime’s (V1.9.1, http://qiime.org/scripts/split_libraries_fastq.html, accessed on 16 April 2022) tags quality control process [31,32]. To obtain Effective Tags, Clean Tags were compared with the species annotation database, and chimeric sequences were removed using vsearch (https://github.com/torognes/vsearch/, accessed on 18 April 2022) [33]. All Effective Tags were clustered into OTUs (Operational Taxonomic Units) using Uparse software (Uparse v7.0.1001, http://www.drive5.com/uparse/, accessed on 19 April 2022) [34] and annotated with species information using the Mothur method with the SSUrRNA database of SILVA132 (http://www.arb-silva.de/, accessed on 19 April 2022) with a threshold of 0.8~1 [35,36]. Phylogenetic relationships were obtained by rapid multiple sequence alignment using MUSCLE software (Version 3.8.31, http://www.drive5.com/muscle/, accessed on 23 April 2022) based on representative sequences in all OTUs. Alpha diversity analysis included Observed species, Chao1, Shannon, Simpson, ACE, Goods-coverage, and PD_whole_tree, which were calculated using Qiime software (Version 1.9.1). Dilution curves, Rank abundance curves, and species accumulation curves were plotted using R software (Version 2.15.3). Beta diversity analysis involved calculating Unifrac distances, constructing a phylogenetic tree based on UPGMA, and performing principal components analysis (PCA), principal co-ordinates analysis (PCoA), and non-metric multi-dimensional scaling (NMDS) plots, all using Qiime software (Version 1.9.1). Intergroup variance analysis of beta and alpha diversity indices was performed using R software with Tukey’s test and Wilcox test of the agricolae package. Finally, linear discriminant analysis effect size (LEfSe) analysis was performed using LEfSe software with a default setting of 4 for the LDA Score filter.

### 2.8. Statistical Analysis

The results were analyzed using GraphPad Prism 5.0 (GraphPad, Inc., La Jolla, CA, USA), and presented as mean ± SD. Statistical analysis to assess differences between groups was performed using the Student’s *t*-test in SPSS 19.0 (IBM Armonk Corp., Armonk, NY, USA), with significance defined as *p* < 0.05. The raw sequence data of the fecal microbiota for this study can be found in the Sequence Read Archive (SRA) database at NCBI under the BioProject ID PRJNA942596.

## 3. Results

### 3.1. Effects of B. subtilis HH2 and ETEC on Beagles

**Figure 1:**
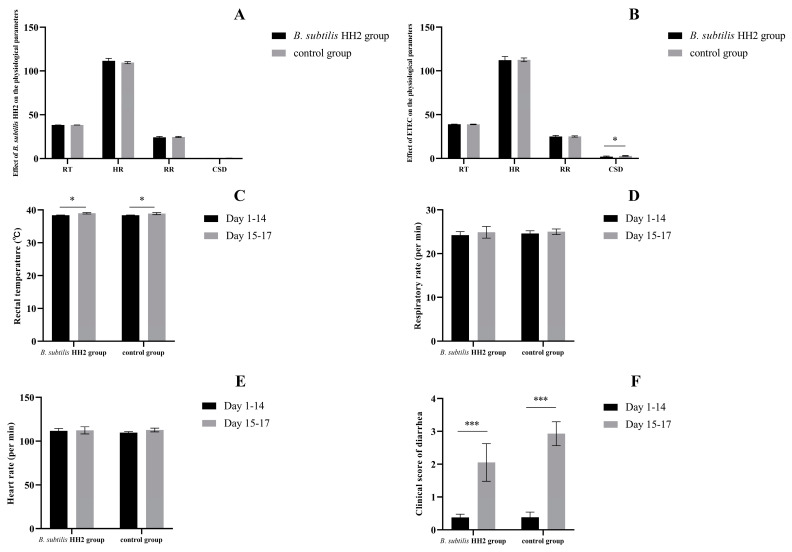
Effects of *Bacillus subtilis* HH2 and enterotoxigenic *Escherichia coli* (ETEC) on rectal temperature (RT), respiratory rate (RR), heart rate (HR), and clinical score of diarrhea (CSD) in beagles: (**A**) Effect of 14-day *B. subtilis* HH2 feeding on physiological parameters of beagles. (**B**) Effect of ETEC on physiological parameters in beagles. Comparison of mean rectal temperature (**C**), mean respiratory rate (**D**), mean heart rate (**E**), and clinical score of diarrhea (**F**) before and after oral ETEC challenge. Statistical analysis using the Student’s *t*-test showed significant differences (* *p* < 0.05; *** *p* < 0.001).

During the 14-day feeding trial, the mean rectal temperature, mean respiratory rate, mean heart rate, and mean inflammatory bowel disease activity index of the dogs in both groups were compared. All dogs in the dogt and dogc groups did not show significant differences in respiratory rate and heart rate during the 14-day feeding period, while none of the dogs showed clinical signs of diarrhea and vomiting (all *p* > 0.05; Figure 1A). After oral administration of ETEC, the mean rectal temperature, mean respiratory rate, and mean inflammatory bowel disease activity index were evaluated in dogs from both groups. All dogs did not show significant treatment needs after the ETEC challenge. After oral administration of ETEC, the two groups did not show significant differences in respiratory rate, rectal temperature, and heart rate (all *p* > 0.05; Figure 1B). Interestingly, the severity of diarrhea was significantly lower in the dogt group than in the dogc group (*p* < 0.05; Figure 1B). Before and after oral administration of ETEC, we assessed the mean rectal temperature, mean respiratory rate, mean heart rate, and mean inflammatory bowel disease activity index in both groups. While there were no significant changes in respiratory rate or heart rate (all *p* > 0.05; Figure 1D,E), all dogs exhibited a significant increase in rectal temperature and severity of diarrhea after receiving ETEC orally compared to their pre-administration values (*p* < 0.05 and *p* < 0.001; Figure 1C,F).

### 3.2. Serum DAO Activity and D-LA Concentration

**Figure 2:**
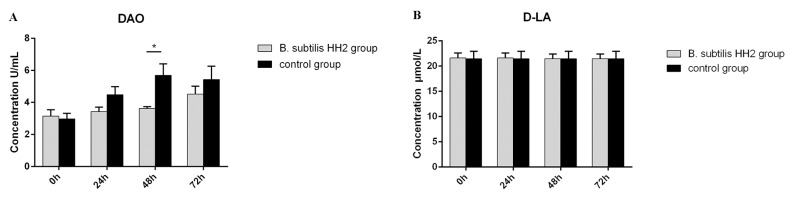
The serum concentration levels of (**A**) diamine oxidase and (**B**) D-lactate in beagles both before and after enterotoxigenic *Escherichia coli* challenge. The results are presented as Mean ± SD of triplicate tests. Statistical analysis using the Student’s *t*-test showed significant differences (* *p* < 0.05).

At 48 h after the ETEC challenge, the dogc group showed significantly higher DAO concentrations compared to the dogt group (*p* < 0.05; Figure 2A). However, there were no significant differences in DAO concentrations between the two groups at 0, 24, and 72 h after the ETEC challenge (*p* > 0.05). Similarly, D-LA concentrations did not differ significantly between the groups during any of the time periods (*p* > 0.05; Figure 2B).

### 3.3. Serum Concentrations of IgG, IgA, and IgM

**Figure 3:**
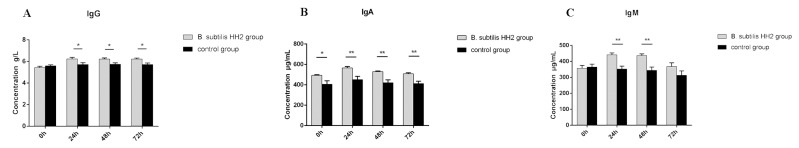
The serum concentration levels of (**A**) IgG, (**B**) IgA, and (**C**) IgM in beagles both before and after enterotoxigenic *Escherichia coli* challenge. The results are presented as Mean ± SD of triplicate tests. Statistical analysis using the Student’s *t*-test showed significant differences (*, *p* < 0.05; **, *p* < 0.01).

The serum levels of IgG, IgA, and IgM after the ETEC challenge are shown in Figure 3. The dogt group had higher IgG at 24, 48, and 72 h after the ETEC challenge than the dogc group (*p* < 0.05; Figure 3A). The dogt group had significantly higher IgA than the control group at all time periods (*p* < 0.05; Figure 3B). The dogt group had significantly higher IgM than the dogc group at 24 and 48 h after the ETEC challenge (*p* < 0.01; Figure 3C).

### 3.4. Assessment of Sequence Data

After splicing the reads, each sample yielded an average of 89,069 raw tags. Following quality control, an average of 81,462 clean tags were obtained, resulting in 60,211 effective tags with an efficiency of 67.63%. These effective tags were then clustered into 6224 OTUs at 97% identity, out of which 1296 (20.82%) were annotated to the genus level using the Silva132 database for identifying their corresponding species. The observed number of OTUs gradually saturated with increasing sequencing depth, indicating that the sequencing depth in this study was sufficient to reflect the microbial diversity (Supplementary Figure S1). The dogt and dogc groups shared a common number of OTUs at 0 h, 24 h, 48 h, and 72 h, which were 1030, 827, 957, and 1011, respectively (Supplementary Figure S2).

### 3.5. Alpha and Beta Diversity Analysis

**Figure 4:**
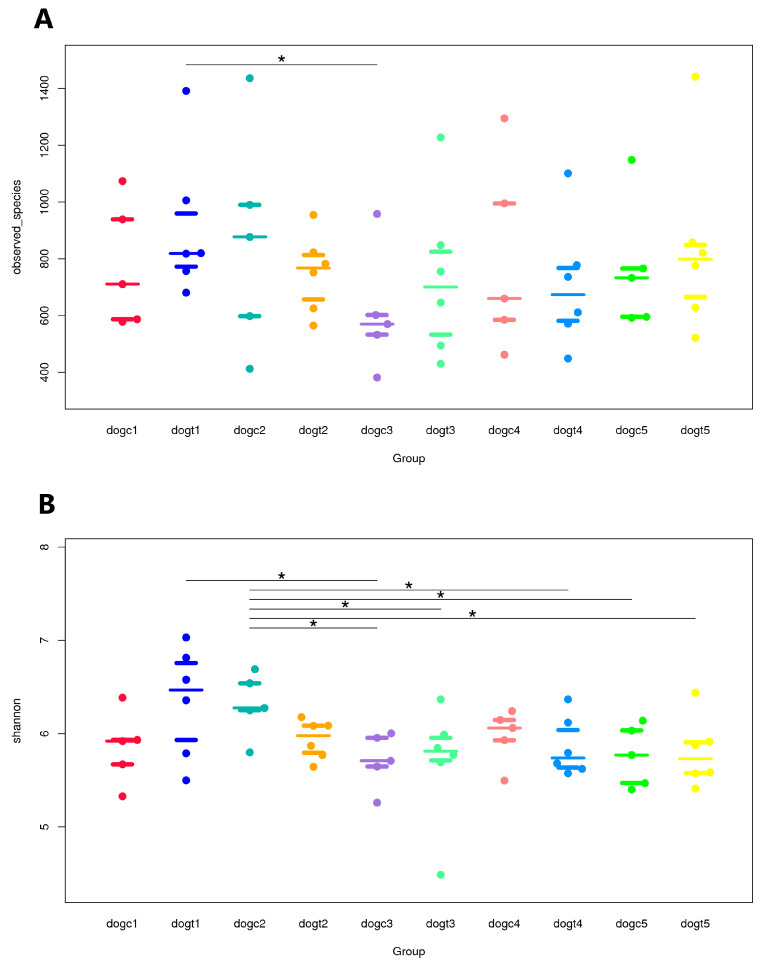
Comparison of alpha diversity of fecal microbiota in the dogt group and the dogc group before and after oral enterotoxigenic *Escherichia coli* (ETEC) challenge: (**A**) Observed species (**B**) Shannon index. Groups (dogt1–dogt5) represent time points in the study: 14 days before and 14 days after *B. subtilis* HH2 feeding, and 24, 48, and 72 h after oral ETEC challenge, respectively. Group (dogc1–dogc5) represents the unfed *B. subtilis* HH2 group. Wilcoxon rank-sum test (*, *p* < 0.05).

Alpha diversity was assessed using five indices: ACE, Chao1, Observed species, Simpson, and Shannon. The ACE, Chao1, and Observed species index are a measure of the number of distinct species that have been detected in the fecal samples of beagles, which is used to estimate the total species richness. Although there was no significant effect on the fecal microbiota in terms of Chao1 and ACE in beagles throughout the experiment, feeding them *B. subtilis* HH2 for 14 days resulted in higher species richness in the fecal sample. Analysis with the Wilcoxon rank-sum test revealed a statistically significant difference in the number of Observed species between the dogt1 and dogc3 groups (*p* = 0.0388 < 0.05), indicating a notable reduction in the abundance of fecal microbiota in beagles not fed *B. subtilis* HH2 at 24 h after oral administration of ETEC (Figure 4A). The Shannon index and Simpson index are measures of species diversity, which reflects differences in species diversity and evenness between samples. Furthermore, the Shannon index was significantly higher in the dogt1 group than in the dogc3 group (*p* < 0.05), suggesting that oral administration of ETEC significantly reduced the gut microbial diversity in the dogc group (Figure 4B). Interestingly, the Shannon and Simpson indices were found to be significantly greater in the dogc2 group compared to the dogc3 and dogc5 groups (all *p* < 0.05; Figure 4B). This indicates that beagles not fed *B. subtilis* HH2 experienced a greater reduction in gut microbial diversity following oral administration of ETEC, compared to beagles in the dogt group. The dogc2 group also had a significantly higher Shannon index than the dogt3 and dogt4 groups, and dogt5. Since they were comparisons of the Shannon index of different groups at different time periods, these comparisons are not included in further analysis.

We compared the microbial community composition of various stool samples using PCA, NMDS, and PCoA based on the weighted UniFrac method. However, we found no clustering among the ten groups (Supplementary Figures S3–S5). To further investigate differences in beta diversity, we performed a Wilcoxon test based on the weighted UniFrac method. The results showed that *B. subtilis* HH2 feeding for 14 days significantly altered the gut microbiota structure of beagles in the dogt group, as indicated by the significant difference in microbial community structure of dogt2 compared to dogt1 and dogc1 (all *p* < 0.001; Supplementary Table S2). Similar results were found for dogt2 with dogt3, dogt4, and dogt5 groups, which shows that oral ETEC challenge altered the fecal bacterial structure at 24, 48, and 72 h after oral administration of ETEC (all *p* < 0.01; Supplementary Table S2). These findings demonstrate that oral administration of ETEC and *B. subtilis* HH2 significantly impacted the gut microbial composition of the dogt group. We also found that the fecal bacterial structure of the dogc group also significantly changed after oral administration of ETEC as compared between dogc1 and dogc5, but this change seemed to be slower compared to the dogt group (*p* < 0.05; Supplementary Table S2).

### 3.6. Community Composition Analysis

**Figure 5:**
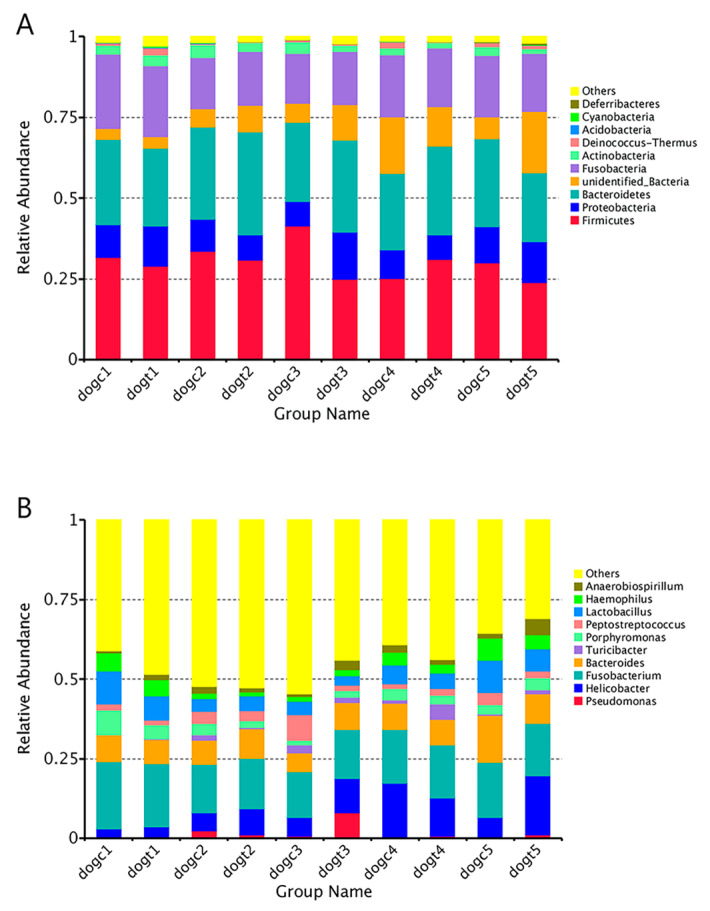
The relative abundance of gut bacterial communities at the top 10 (**A**) phyla and (**B**) genera. Groups (dogt1–dogt5) represent time points in the study: 14 days before and 14 days after *Bacillus subtilis* HH2 feeding, and 24, 48, and 72 h after oral enterotoxigenic *Escherichia coli* challenge, respectively. Group (dogc1–dogc5) represents the unfed *B. subtilis* HH2 group.

**Figure 6:**
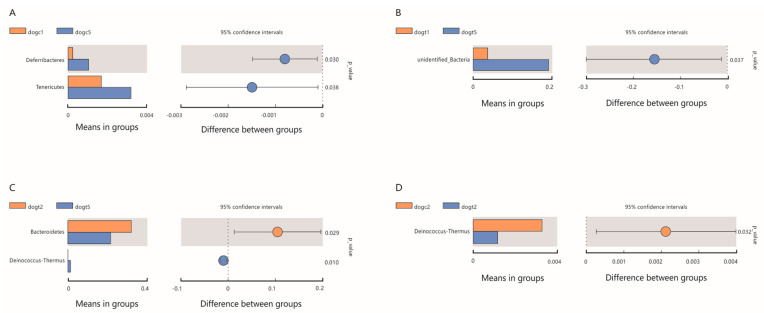
Comparison of species with significant differences between groups at the phylum level (*t*-test): (**A**) Comparison of significantly different species pre− and post−72 h oral enterotoxigenic *Escherichia coli* (ETEC) challenge in the dogc group. (**B**) Comparison of significantly different species before feeding *B. subtilis* HH2 (dogt1) and 72 h after oral ETEC challenge (dogt5) in the dogt group. (**C**) Comparison of significantly different species after 14 days of *B. subtilis* HH2 feeding (dogt2) and 72 h after oral ETEC challenge (dogt5) in the dogt group. (**D**) Comparison of significantly different species between the dogc and dogt groups after 14 days of *B. subtilis* HH2 feeding.

We created a cumulative bar chart using the relative abundance of the top 10 species identified at each taxonomic level of phylum and genus. The chart revealed that *Firmicutes* (30.14 ± 5.14%), *Bacteroidetes* (26.39 ± 3.00%), and *Fusobacteria* (18.35 ± 2.53%) were the most abundant phyla among all groups, followed by *Proteobacteria* (10.26 ± 2.46%) (Figure 5A). At the genus level, *Fusobacterium* (16.9 ± 2.0%) and *Helicobacter* (9.1 ± 5.2%) dominated, followed by *Bacteroides* (8.8 ± 2.2%) in all groups (Figure 5B). Heat maps of the 30 richest bacterial communities at the phylum and genus levels demonstrated both similarities and differences between the 10 groups (Supplementary Figures S6 and S7). To detect species that differed significantly between groups, we performed T-tests and LEfSe analysis to identify biomarker species. In the dogc group, the proportion of *Deferribacteres* and *Tenericutes* in feces was significantly higher at 72 h after oral ETEC administration compared to before administration (*p* < 0.05; Figure 6A). In contrast, in the dogt group, the proportion of unidentified bacteria in feces was significantly higher at 72 h after oral ETEC administration compared to before oral administration of *B. subtilis* HH2 (*p* < 0.05; Figure 6B). Interestingly, in the dogt group, the proportion of *Bacteroidetes* in feces was significantly higher after 14 days feeding of *B. subtilis* HH2 compared to 72 h after administration of ETEC (*p* < 0.05; Figure 6C). The proportion of *Deinococcus*-*Thermus* in feces was significantly higher at 72 h after oral ETEC administration than the results after 14 days of feeding *B. subtilis* HH2 (*p* < 0.05; Figure 6C). At the end of 14 days of feeding *B. subtilis* HH2, *Deinococcus*-*Thermus* in feces of the dogt group was significantly lower than that of the dogc group (*p* < 0.05; Figure 6D). We further analyzed the relative abundance of gut microbiota composition at the genus in the dogt and dogc groups. We found that after two weeks of feeding *B. subtilis* HH2, the abundance of *Thermus* was significantly lower compared to the dogc group (*p* < 0.05; Supplementary Figure S8). The abundance of *Romboutsia* and *Butyricicoccus* in the dogt group was significantly higher than in the dogc group at 72 h after oral administration of ETEC (*p* < 0.05; Supplementary Figure S9).

**Figure 7:**
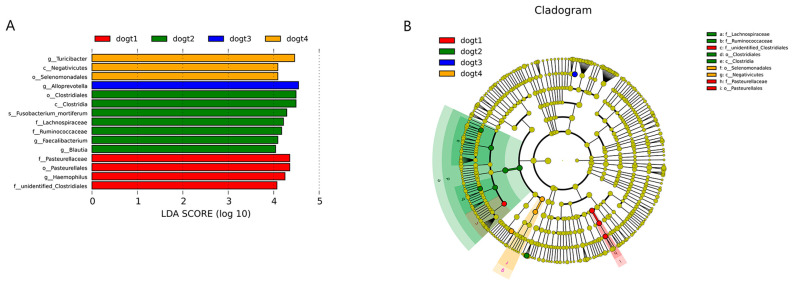
Statistically different bacterial taxa in feces at different time points in the dogt group: (**A**) Bacterial taxa with significant differences in abundance in different groups are shown, and the length of the bar chart represents the magnitude of the impact of different species (LDA Score > 4). (**B**) Cladogram shows the taxonomic hierarchy of bacterial taxa that are significantly abundant in groups.

Finally, we found significant differences in the abundance of species in the dogt group by LEfSe analysis. *Clostridiales* (order), *Clostridia* (class), *Fusobacterium_mortiferum* (species), *Lachnospiraceae* (family), *Ruminococcaceae* (family), *Faecalibacterium* (genus), and *Blautia* (genus) were more abundant after oral administration of *B. subtilis* HH2 in the dogt group (Figure 7). In contrast, *Turicibacter* (genus), *Negativicutes* (class), and *Selenomonadales* (order) were enriched at 48 h after oral ETEC administration in the dogt group (Figure 7). In the dogt group, there were also species with statistical differences before feeding *B. subtilis* HH2 and 24 h after oral administration of ETEC.

## 4. Discussion

To address the issue of antibiotic resistance, probiotics have emerged as a promising strategy for enhancing gut health in hosts. Studies have demonstrated the efficacy of probiotics, including *Lactobacillus*, *Bifidobacterium*, and *Bacillus*, in preventing bacterial infections in the gut [37,38,39]. In our previous research, we isolated *B. subtilis* strain HH2 from the feces of a healthy giant panda, which showed promising results in acting as a probiotic for pandas on a high-fiber diet, inhibiting *E. coli* and *Staphylococcus aureus* in vitro, and ameliorating TNBS-induced colitis [23,24,25]. The objective of this study was to evaluate the protective effects of *B. subtilis* HH2 in beagles challenged with ETEC. We assessed beagles by measuring their serum concentrations of IgG, IgA, and IgM, levels of markers of intestinal barrier (DAO and D-LA), and the impact of *B. subtilis* HH2 on gut microbiota before and after the ETEC challenge.

Fourteen days of *B. subtilis* HH2 feeding did not alter the basic physiological indicators and did not cause digestive symptoms, indicating that *B. subtilis* HH2 is safe for dogs and worthy of further clinical application. Although both groups experienced increased body temperature, anorexia, and mild diarrhea after oral ETEC, feeding *B. subtilis* HH2 significantly alleviated gastrointestinal symptoms caused by ETEC. Xu et al. also reported similar observations that probiotic feeding reduced the incidence of diarrhea in dogs [40,41].

The stability of the mucosal environment in the gut is heavily reliant on the integrity of the intestinal tract barrier, which acts as a crucial link between gut microbes and the immune system in the intestines. When the intestinal mucosa is damaged or the tight junctions between cells are disrupted, D-LA produced by bacteria and DAO released by damaged intestinal mucosal cells enter the bloodstream, leading to an increase in serum D-LA levels and DAO activity. Therefore, monitoring the blood levels of D-LA can serve as an effective measure to timely detect the degree of intestinal mucosal damage and permeability, while DAO levels can indicate the functional status of the intestinal mucosa. Reports indicate that ETEC invasion increases DAO and D-LA release into the plasma, damaging the intestinal epithelial cell membrane [42,43]. In the present study, we observed a significant decrease in plasma DAO levels at 48 h after ETEC infection in the dogt group, consistent with a prior study where the supplementation of diets with *B. subtilis* reduced plasma DAO activity in piglets with intrauterine growth restriction [44]. Nevertheless, no significant difference was observed in serum levels of D-LA compared to the dogc group, suggesting that the beagle model of infection with ETEC may not be fully established. Although no significant differences in serum levels of D-LA were observed between the groups, the reduction in destruction of intestinal epithelial cells by ETEC was evident in the change in DAO serum levels, indicating the efficacy of *B. subtilis* HH2 feeding. For future studies, more detection metrics should be included to support the successful construction of the model. Our results, for the first time in dogs, confirm the protective effect of this strain in strengthening the intestinal barrier during ETEC invasion.

Various immunoglobulins are present in the serum and have different biological functions. Among them, IgA plays a crucial role in the humoral adaptive immune system, particularly at mucosal sites. Studies have found that oral probiotics boost the number of IgA^+^ cells in the lamina propria of various organs, including the intestines, bronchus, and mammary glands [45,46]. Additionally, probiotics have been shown to induce Th1 balance that promotes the production of IgG [47]. While some studies have suggested that probiotics may have an immunomodulatory effect, the evidence is not clear that probiotics stimulate elevated serum IgM levels. According to previous studies, *Clostridium butyricum, Lactobacillus*, and *B. subtilis* enhanced serum IgG, IgM, and IgA levels, which suggests that probiotics may stimulate systemic or mucosal responses [48,49,50]. The supplementation of diets with *B. subtilis* has also been found to promote the development of immune organs [49]. In the present study, *B. subtilis* HH2 clearly increased IgG, IgM, and IgA levels before or after the ETEC challenge. This may be the result of *B. subtilis* HH2 interacting with the host and gut microbiota. However, there are also strains that have no effect on the serum immunoglobulins of IgG, IgA, and IgM [51]. A study found that piglets fed a diet containing high-dose *B. subtilis* experienced a reduction in serum IgG and IgM levels [52]. To better understand the potential of *B. subtilis* in increasing serum immunoglobulin levels, further research is imperative to explore the underlying mechanisms.

Probiotics refer to live microorganisms that can provide health benefits to the host when consumed in sufficient quantities. Probiotics are thought to modulate the gut microbiota composition and function, resulting in a favorable microbial balance [53]. Moreover, probiotics have been shown to have beneficial effects in the prevention and treatment of a range of digestive disorders, such as irritable bowel syndrome [54], inflammatory bowel disease [55], and antibiotic-associated diarrhea [56]. ETEC challenge can cause individual-specific changes in gut microbiota [57], leading to a lower bacterial richness and altered microbial composition [58]. Recent research suggests that probiotic *B. subtilis* may help mitigate the effects of ETEC infection by modulating the gut microbiota [59]. Microbial richness was increased after 14 days feeding of *B. subtilis* HH2 but decreased at 24 h after the ETEC challenge in the dogt group, suggesting that feeding *B. subtilis* HH2 could increase the fecal bacterial richness and ETEC challenge reduced microbial richness in feces. However, the dogc group that was fed commercial dog food for 14 days also showed an increase in fecal microbiota richness, consistent with previous research demonstrating the impact of diet on the fecal microbiota of dogs [60]. Future studies will need to reconsider the composition of the dog’s diets to minimize the effect on the experimental results. Notably, the dogc group showed a significant decrease in species richness and diversity at 24 h after oral administration of ETEC, indicating that ETEC may cause more severe disorders of the gut microbiota than the dogt group. Beta diversity is a measure of the variation in species composition between different samples. However, the lack of clustering in the stool samples from both groups suggests that the impacts of ETEC or *B. subtilis* HH2 on fecal microbial community composition may be limited, or that commercial dog food could also have influenced the fecal microbial community composition. Similar to studies on weaned pigs, alpha and beta diversity analyses also suggest that dietary supplementation of *B. subtilis* has a limited impact on the fecal microbiota in fecal samples [18]. The composition and structure of gut microbes in animals vary during different developmental stages, highlighting the lack of rigor in comparing fecal microbiota at different time points in this study. Hence, for future studies, it is imperative to incorporate a control group that remains untreated and intervention-free to ensure scientific validity.

In this study, *Firmicutes* and *Bacteroidetes* are the predominant gut bacterial communities of beagles, which is consistent with a previous study [61]. Our results indicate that after 14 days of feeding *B. subtilis* HH2 to beagles, the relative abundance of *Bacteroidetes* and unidentified bacteria increased greatly, while the relative abundance of *Proteobacteria* decreased when compared to the dogc group. These findings suggest that feeding *B. subtilis* HH2 may have a beneficial effect on the gut health of beagles, possibly due to the ability of *Bacteroidetes* to break down carbohydrates, proteins, and other substances, thereby enhancing nutrient utilization in the host’s body [62]. An overabundance of *Proteobacteria* has been linked to dysbiosis in individuals who have metabolic or inflammatory conditions [63]. Moreover, previous studies have shown that the ETEC challenge increased the abundance of *Proteobacteria* and *Firmicutes,* and reduced the abundance of *Bacteroides* [18,64]. Similarly, in the dogc group, we observed a significant increase in the relative abundance of *Firmicutes* and a decrease in the relative abundance of *Bacteroides* in the stool at 24 h after the oral administration of ETEC. Furthermore, in the dogt group, there was also an increase in the relative abundance of *Proteobacteria* in the feces at 24 h after the oral ETEC challenge. Bacterial community comparisons indicated 14 days feeding of *B. subtilis* HH2 significantly reduced the abundance of *Deinococcus*-*Thermus*. While *Deinococcus*-*Thermus* is not commonly found in the gut microbiota, some studies have suggested that *Deinococcus*-*Thermus* bacteria were more prevalent in the gut microbiota of individuals with Graves’ orbitopathy patients than in those with Graves’ disease [65]. Therefore, future studies should further explore the correlation between *Deinococcus*-*Thermus* and *B. subtilis* HH2.

According to previous studies, the effectiveness of most probiotics in animals has been demonstrated with a daily consumption of microorganisms ranging from 10^7^ to 10^9^ [26,66]. In the present study, daily oral intake of 5.0 × 10^9^ *B. subtilis* HH2 protected the gut of beagles against the ETEC challenge. However, to establish the optimal dosage, future studies should consider incorporating different dose groups and drug treatment groups to further investigate the potential effects of varying interventions, as the current experimental design did not allow for such analysis. Overall, our findings suggest that the probiotic *B. subtilis* HH2 has a positive effect against ETEC by increasing bacterial community richness and diversity, improving immunity, and enhancing intestinal barrier function. These findings indicate that *B. subtilis* could be used as a dietary supplement or therapeutic agent to prevent or alleviate ETEC-induced gut dysbiosis in veterinary medicine.

## Data Availability

The raw sequence data of the fecal microbiota for this study can be found in the Sequence Read Archive (SRA) database at NCBI under the BioProject ID PRJNA942596.

## Supplementary Materials

The following supporting information can be downloaded at: https://www.mdpi.com/article/10.3390/vetsci10070432/s1, Figure S1: Rarefaction curve; Figure S2: Venn Graph; Figure S3: Principal Component Analysis; Figure S4: Principal Co-ordinates Analysis based on Weighted Unifrac distance; Figure S5: Non-Metric Multi-Dimensional Scaling analysis; Figure S6: Heatmap of the 30 most abundant phylum among different groups; Figure S7: Heatmap of the 30 most abundant genera among different groups; Figure S8: Comparison of species with significant differences between groups at the genus level (dogc2–dogt2); Figure S9: Comparison of species with significant differences between groups at the genus level (dogc5–dogt5); Table S1: Product nutrition analysis value; Table S2: Results of inter-group differences analysis of beta diversity based on Wilcoxon rank-sum test.
