# Supplementary material for: Protective Effects of Bacillus subtilis HH2 against Oral Enterotoxigenic Escherichia coli in Beagles
Source: Vet Sci. 2023 Jul 3;10(7):432. doi: 10.3390/vetsci10070432 (PMC10384286; doi:10.3390/vetsci10070432)
Supplement: Supplementary file 1 [file vetsci-10-00432-s001.zip › vetsci-2377246-supplementary.pdf]

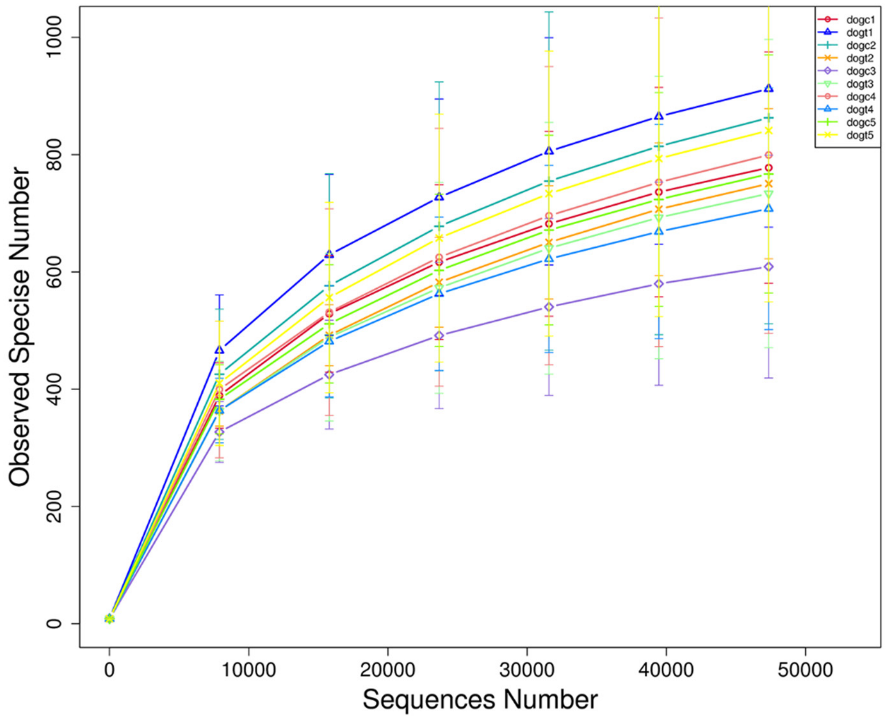

Supplementary Figure S1. Rarefaction curve. The horizontal axis represents the number of sequencing reads randomly sampled from a certain sample, and the vertical axis represents the number of OTUs that can be constructed based on this sequencing read count, which reflects the sequencing depth. Different samples are represented by curves of different colors.

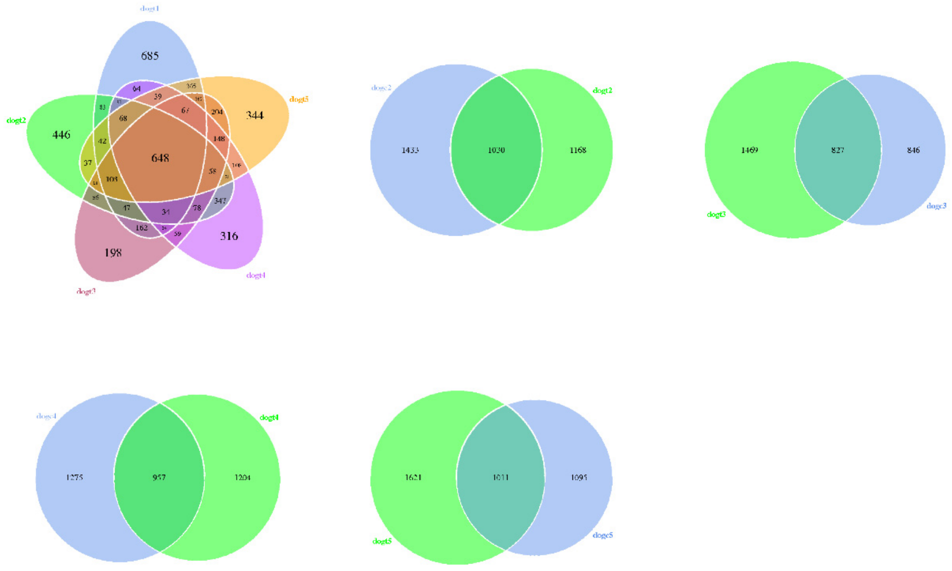

Supplementary Figure S2. Venn Graph. Each circle in the figure represents a (group) sample, and the number in the overlapping part of the circles represents the number of

OTUs shared between the samples (groups). The number in the non-overlapping part represents the number of unique OTUs in each sample (group).

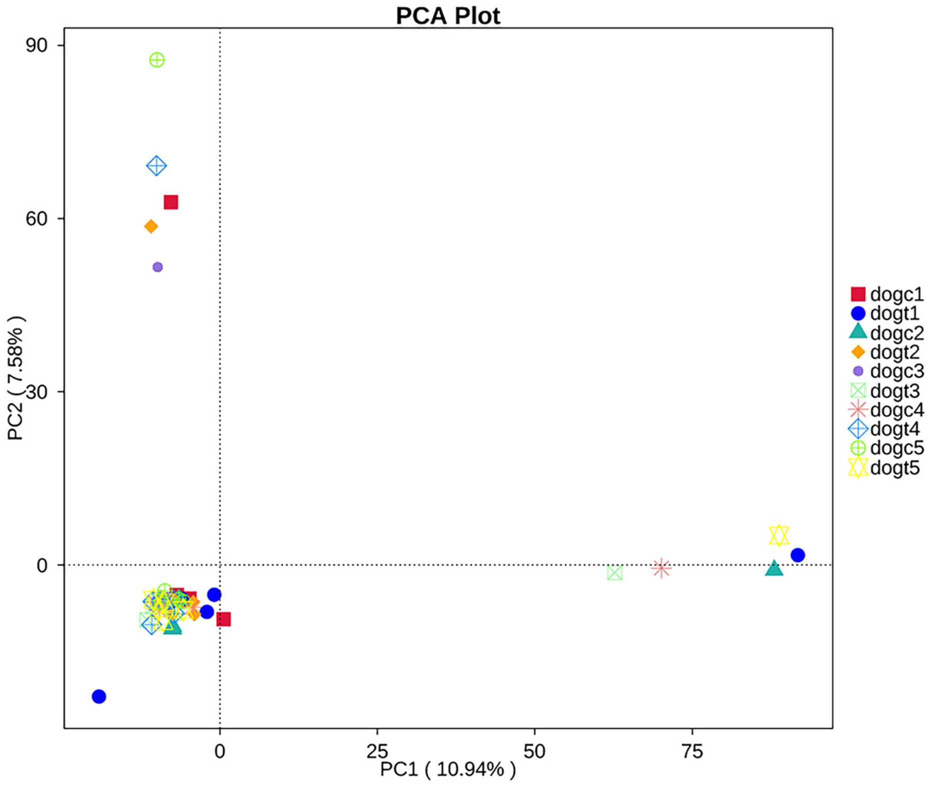

Supplementary Figure S3. Principal Component Analysis. The horizontal axis represents the first principal component, and the percentage represents the contribution of the first principal component to the differences between the samples. The vertical axis represents the second principal component, and the percentage represents the contribution of the second principal component to the differences between the samples. Each point in the figure represents a sample, and samples from the same group are represented in the same color.

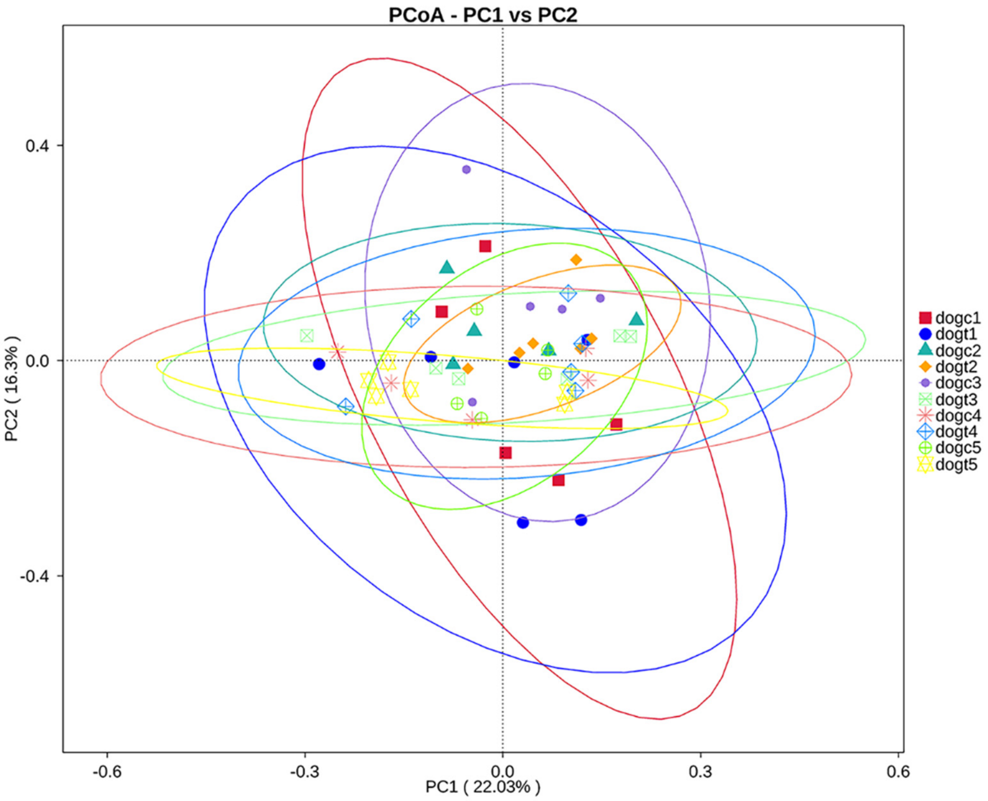

Supplementary Figure S4. Principal Co-ordinates Analysis based on Weighted Unifrac distance. The horizontal axis represents one principal component, and the vertical axis represents another principal component. The percentage represents the contribution of the principal component to the differences between the samples. Each point in the figure represents a sample, and samples from the same group are represented in the same color.

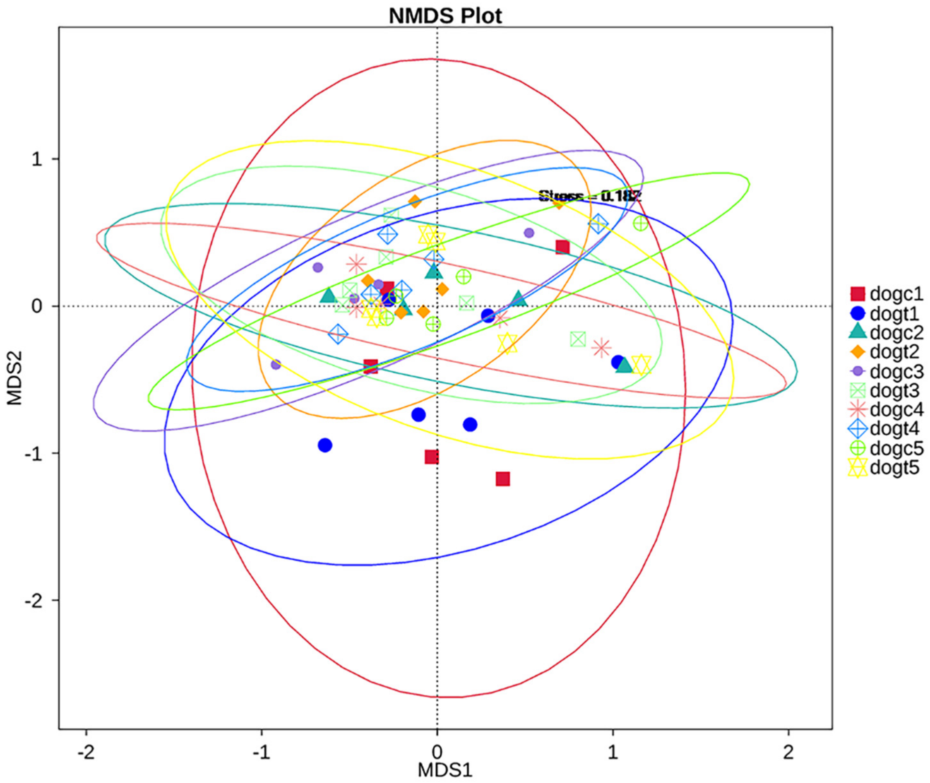

Supplementary Figure S5. Non-Metric Multi-Dimensional Scaling analysis. Each point in the figure represents a sample, and the distance between points represents the degree of difference between them. Samples from the same group are represented in the same color. When the stress value is less than 0.2, it indicates that NMDS can accurately reflect the degree of difference between samples.

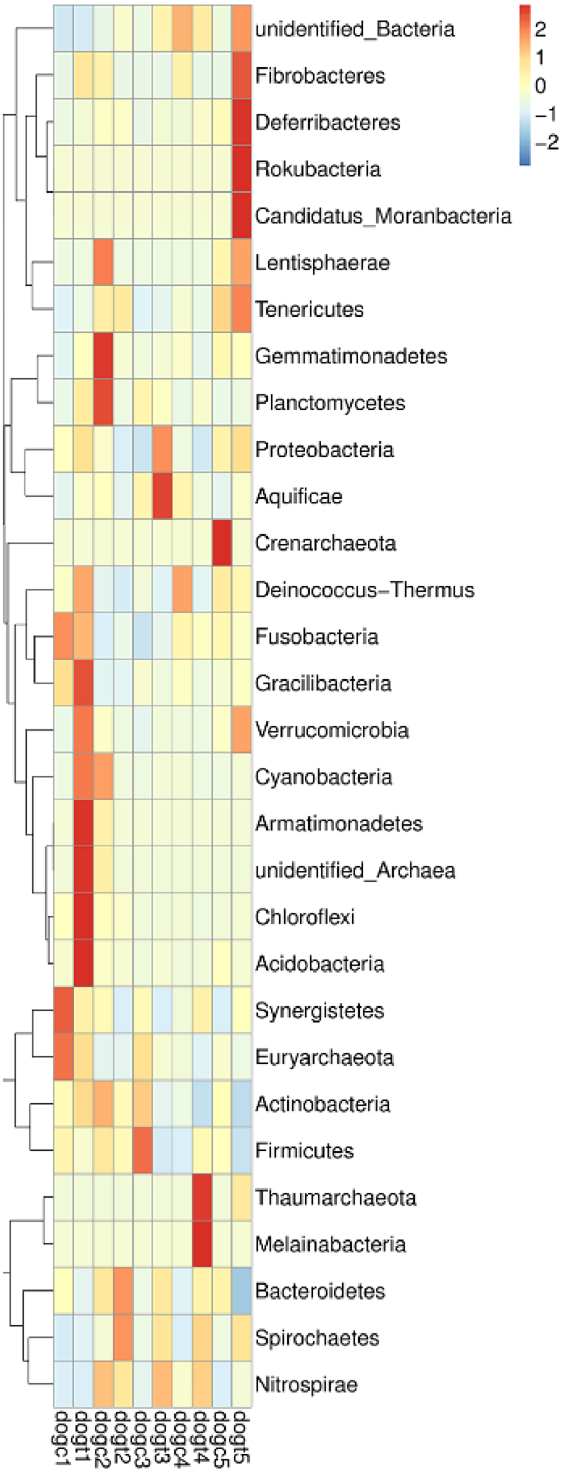

Supplementary Figure S6. Heatmap of the 30 most abundant phylum among different groups. The horizontal axis represents sample information, and the vertical axis represents species annotation information.

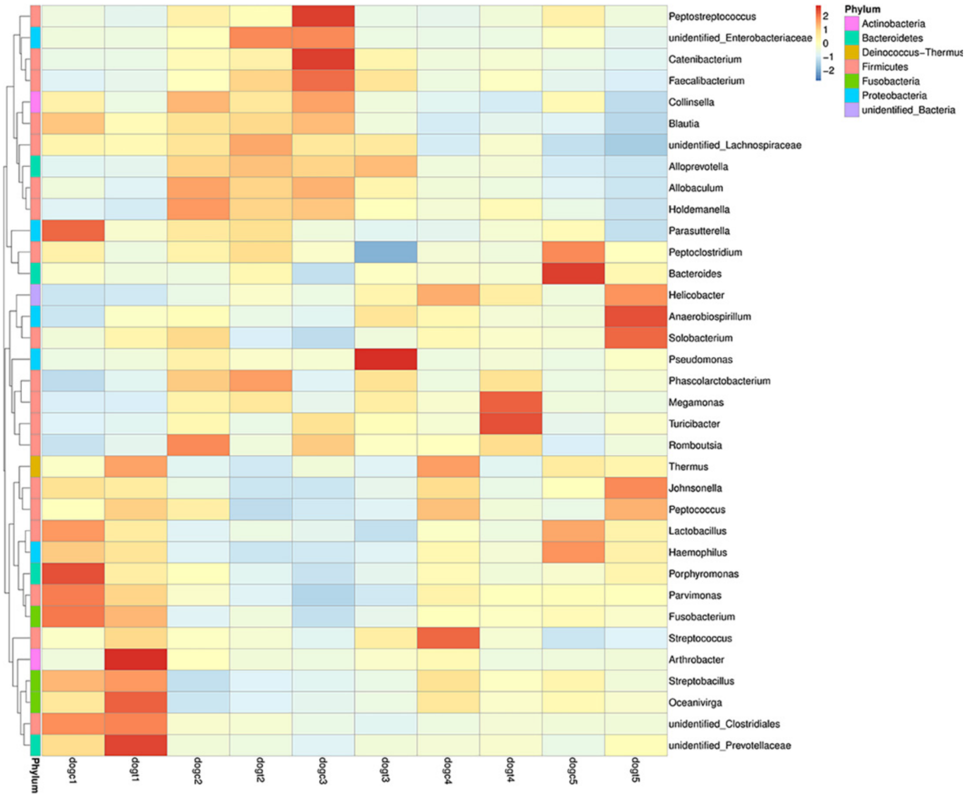

Supplementary Figure S7. Heatmap of the 30 most abundant genera among different groups. The horizontal axis represents sample information, and the vertical axis represents species annotation information.

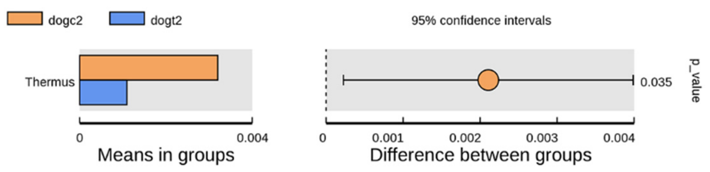

Supplementary Figure S8. Comparison of species with significant differences between groups at the genus level (dogc2-dogt2). Statistically significant differences (P value < 0.05) were found between the two main groups using T-test.

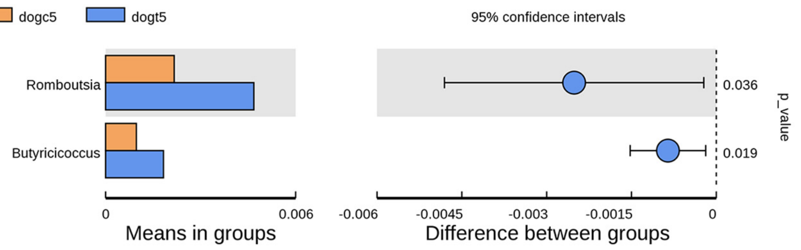

Supplementary Figure S9. Comparison of species with significant differences between groups at the genus level (dogc5-dogt5). Statistically significant differences (P value < 0.05) were found between the two main groups using T-test.

Nutrient	Value (%)
Crude protein (min)	⩾20%
Crude fat (min)	⩾10%
Crude fiber (max)	⩽5.0%
Crude ash content (max)	⩽8.5%
Moisture content (max)	⩽11%
Calcium (min)	⩾1.2%
Total phosphorus (min)	⩾0.8%
Mineral (min)	⩾1.5%
Lysine (min)	⩾1.2%
Omega-3 (min)	⩾0.8%

Supplementary Table S1. Product nutrition analysis value. The main compositions of the dog food include beef, fruits and vegetables, coarse grains, vitamin A, vitamin B, vitamin D, fish oil, minerals. The nutrition analysis value and composition of the dog food are provided by the manufacturer.

Group	Difference	p-value	sig.	LCL	UCL
dogc1 – dogt2	55.4	1e−04	***	28.7305900186046	82.0694099813955
dogt1 – dogt2	63.2	0	***	39.3461545447154	87.0538454552846
dogt2 – dogt3	−40.0666666666667	0.0012	**	−63.9205121219513	−16.212821211382
dogt2 –	−38.5333333333333	0.0018	**	−62.387178788618	−14.6794878780487

dogt4					
dogt2 – dogt5	−38.2666666666667	0.0019	**	−62.1205121219513	−14.412821211382

Supplementary Table S2. Results of inter-group differences analysis of beta diversity based on Wilcoxon rank-sum test. Difference: Mean difference; sig.: Whether it is significant or not, if p value <0.01, mark **, p value <0.001, ***; LCL： Lower limit of confidence interval; UCL： Upper limit of confidence interval.
